# A Systematic Mapping Review of Core Outcome Reporting in Surgical Research for Oesophageal Cancer

**DOI:** 10.1002/jso.70173

**Published:** 2026-01-07

**Authors:** Nadia Matias, Anie Naqvi, Jack Thomson, Roukia Techache, Kerry Avery, Natalie Blencowe, Rhiannon Macefield, Bilal Alkhaffaf

**Affiliations:** ^1^ Department of Oesophago‐Gastric and Bariatric Surgery, Salford Royal Hospital Northern Care Alliance, Stott Lane Salford UK; ^2^ Centre for Surgical Research, and NIHR Bristol Biomedical Research Centre Population Health Sciences, Bristol Medical School, University of Bristol Bristol UK; ^3^ Faculty of Biology, Medicine and Health University of Manchester Oxford Rd Manchester UK

**Keywords:** cancer, core outcomes, oesophageal, surgical resection

## Abstract

Oesophageal carcinoma is a rising global health burden, with surgical resection and perioperative chemotherapy forming the cornerstone of curative treatment. However, uncertainty persists regarding the optimal surgical approach, partly due to heterogeneity in outcome reporting, which hinders data synthesis and evidence‐based decision‐making. To address this, a core outcome set (COS) for oesophageal cancer surgery was developed through international consensus among clinicians and patients. This study systematically evaluates the uptake of these core outcomes in contemporary surgical research. A systematic review was conducted of randomised controlled trials and prospective cohort studies investigating oesophagectomy for oesophageal cancer, published between 2010 and 2024. The reporting of ten COS‐recommended outcomes was assessed across eligible studies. Fifty‐eight studies involving 22 260 patients were included (39 cohort studies; 19 RCTs). No study reported all 10 core outcomes. The median number of core outcomes reported was 4 (interquartile range 3–5). The frequency of individual core outcome reporting was as follows: in‐hospital mortality (86%), conduit necrosis/leak (81%), respiratory complications (79%), overall survival (30%), ability to eat and drink (44%), quality of life (26%), inoperability (23%), reflux symptoms (21%), severe nutritional effects (19%), and need for reintervention (16%). No improvement in core outcome reporting was observed over the study period. Promoting COS implementation and improving methodological rigour is essential to ensure that future research reflects the priorities of both clinicians and patients, and facilitates meaningful evidence synthesis.

**Abbreviations**
COMET: the core outcome measures in effectiveness trials
COS: core outcome set
PRISMA: preferred reporting items for systematic reviews and meta‐analyses
RCT: randomised controlled trial

## 1 Introduction

Oesophageal cancer remains a significant health burden as the eighth most common malignancy worldwide, with an increasing incidence in the Western world [1].

Although there has been significant improvement in the survival of localised oesophageal cancer [2], surgery post neoadjuvant therapy remains the cornerstone of curative treatment in locally advanced tumours [3]. Minimally invasive surgery for oesophageal cancer was first introduced in 1992. The long‐term results of the first randomised controlled trial (RCT) comparing hybrid techniques (laparoscopic abdominal phase and open thoracic phase) to open surgery were published in 2019 [3]. There have been few comprehensive RCTs comparing resection techniques based on postoperative outcomes. This may be due to the complexities of designing and delivering a surgical RCT, not least due to lack of standardisation in surgical expertise among various centres and strong surgeon preferences, which makes recruitment challenging [4].

Patients and clinicians have differing priorities on outcomes and what should be reported in the literature [5]. This heterogeneity poses a challenge to the systematic assessment of surgical approaches, contributing to significant research waste [6].

A core outcome set (COS) [6] was developed following a rigorous consensus process based on the COMET guidelines [7]. This was primarily undertaken to guide the development and application of a “minimum set of outcomes that should be measured and reported in all clinical trials of a specific disease or trial population” [7]. In the case of oesophageal cancer resection, the following 10 core outcomes were classed as essential: overall survival, in‐hospital mortality, inoperability, need for additional intervention, respiratory complications, conduit necrosis and anastomotic leak, severe nutritional problems, the ability to eat and drink, problems with acid indigestion or heartburn, and overall quality of life.

This study aims to map the reporting of core outcomes included in the previously described COS and establish the heterogeneity in more recent studies evaluating surgical treatments for oesophageal cancer. This would highlight the degree of uptake of the COS and identify areas requiring future work to improve the status quo.

## 2 Methods

This review was registered on the PROSPERO database and reported in line with PRISMA (Preferred Reporting Items for Systematic Reviews and Meta‐Analyses) guidance [8].

### 2.1 Definitions

This review focuses on surgical studies comparing two or more therapeutic surgical interventions [9]. We define ‘therapeutic surgical intervention′, in the context of oesophageal malignancy, as a potentially curative intervention aiming to excise the oesophageal neoplasm, resulting in partial or total organ loss. Therefore, non‐surgical (e.g., endoscopic) therapies were excluded from this study.

### 2.2 Search Strategy

An initial systematic review included studies published from January 2006 to May 2011 [6] in conjunction with the development of the COS. This was used to aid the identification of trials for inclusion in the present study. An updated search strategy was generated between January 2010 and October 2024. The strategy is described in the supplementary *appendix 1*. The following databases were searched for studies that met the inclusion criteria

- Evidence Based Medicine Reviews via OVID (1 January 2010 to 1 October 2024)
- Cochrane Database of Systematic Reviews
- ACP Journal Club
- Database of Abstracts of Reviews of Effects
- Cochrane Central Register of Controlled Trials
- Health Technology Assessment
- NHS Economic Evaluation Database
- MEDLINE via OVID, EMBASE via OVID, CINAHL via EBSCO (1 January 2010 to 1 October 2024)

### 2.3 Eligibility Criteria

The inclusion and exclusion criteria are summarised in Table 1. Systematic reviews were included to identify relevant RCTs from the reference list. Non‐English language studies were excluded, as were conference abstracts, as word count limitations would likely restrict reporting of all outcomes measured in a study.

**Table 1:** Inclusion and exclusion criteria.

	Inclusion	Exclusion
Type of studies	RCTs Prospective cohort studies or cohort studies with prospectively maintained databases English language studies	Surgical RCTs, systematic reviews and cohort studies involving combination of surgical and non‐surgical interventions Retrospective cohort studies Trial protocols Non‐English language studies
Population	Patients aged 18 years and over	Patients below the age of 18
Intervention	Partial or total oesophagectomy Surgery with curative intent Oesophagectomy for gastro‐oesophageal junctional tumours	Surgery with non‐curative intent (i.e., in stage 4 cancer, expected R1/R2 resection), for example for relief of symptoms Salvage surgery Endoscopic interventions Partial or total gastrectomy
Conditions	Invasive localised cancer of oesophagus and gastro‐oesophageal junction Surgically resectable tumours	Barret′s dysplasia or non‐invasive oesophageal neoplasms Metastatic/advanced disease Sarcomas Lymphomas

*Note:* Inclusion and exclusion criteria of all included studies.

### 2.4 Data Analysis

Our group has previously described the heterogeneity of outcome reporting in the present published literature [6]. A meta‐analysis is beyond the scope of this review, as it primarily looks at the quality of ‘what′ and ‘how′ outcomes are reported. Descriptive statistical analysis was performed using Microsoft Excel. Statistical analysis using two‐tailed Fisher's Exact Test was performed on the cumulative core outcomes, offering a comparison pre and post‐publication of the COS.

## 3 Results

### 3.1 Study Selection

**Figure 1:**
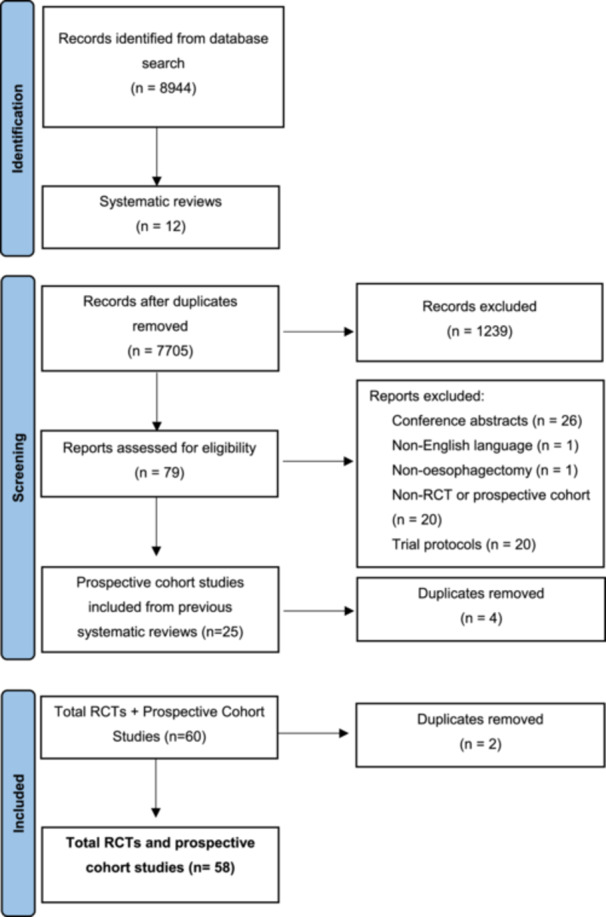
Preferred reporting items for systematic reviews and meta‐analyses (PRISMA) flowchart summarising study selection process.

There were 8944 studies identified from the initial database search, which included primary studies and systematic reviews. After resolving discrepancies, 79 articles were included for analysis. These included 12 systematic reviews to source studies that may have been missed in the initial search (Figure 1). 58 individual studies were included for analysis. Articles reporting the same study were analysed together for ease of reference and to avoid duplication.

### 3.2 Study Characteristics

The 58 studies that met the inclusion criteria included 19 RCTs and 39 prospective cohort studies (Table 2‐ *Appendix*). The majority of studies were undertaken in Europe and North America (64%) and the rest in Asia (33%), with one study in Australia. A total of 22 260 patients undergoing oesophageal resections for cancer were recruited. The smallest number of patients in a single study was 13, and the largest was 5822. The largest number of studies on oesophageal cancer resections was published in 2019. Most (24.6%) studies, published between 2010 and 2012, compared the outcomes of variations of minimally invasive and hybrid techniques with open surgery, after which the results of the first trial of robot‐assisted oesophagectomy were published in 2012 [12]. Four studies primarily evaluated the effect of lymphadenectomy on outcomes.

**Table 2:** Included studies.

Randomised controlled trials							
Study	Author	Publication year	Countries	Recruiting centres	Randomised patients	Interventions	Type of Study
PRE‐ COS							
1	YC Yuan et al. [10]	2011	China	1	68	– Modified thoracoscopic oesophagectomy – Minimally invasive oesophagectomy	RCT
2	Biere et al. [11]	2012	Netherlands, Spain, Italy	3	105	– Minimally invasive oesophagectomy – Open oesophagectomy	RCT
3	Van der Sluis et al. [12]	2012	Netherlands	1	112	– Robot‐assisted thoraco‐laparoscopic oesophagectomy – Open three‐stage transthoracic esophagectomy	RCT
4	Guo et al. [13]	2013	China	1	221	– Video‐assisted thoracoscopic radical esophagectomy (VATS) combined with laparoscopy – Right open transthoracic oesophagectomy	RCT
5	Maas et al. [14]	2014	Netherlands	1	27	– Minimally invasive oesophagectomy – Open oesophagectomy	RCT
6	Li et al. [15]	2015	China	1	300	– Ivor Lewis oesophagectomy – Sweet oesophagectomy	RCT
7	Luketich et al. [16]	2015	USA	1	95	– 3 stage minimally invasive oesophagectomy – 2 stage minimally invasive oesophagectomy	RCT
8	Nozaki et al. [17]	2015	Japan	37	379	– Open oesophagectomy – Thoracoscopic oesophagectomy	RCT and Prospective Cohort
9	Straatman et al. [18]	2017	Netherlands, Spain, Italy	3	115	– Minimally invasive oesophagectomy – Open oesophagectomy	RCT
POST COS							
10	Ma et al. [19]	2018	China	1	144	– Minimally invasive oesophagectomy – Open oesophagectomy	RCT
11	Paireder et al. [20]	2018	Austria	1	29	– Open oesophagectomy – Hybrid minimally invasive oesophagectomy	RCT
12	Mariette et al. [1]	2019	France	13	207	– Open oesophagectomy – Hybrid minimally invasive oesophagectomy	RCT
13	Van der Sluis et al. [21]	2019	Netherlands	1	112	– Robot‐assisted thoraco‐laparoscopic esophagectomy – Open three‐stage transthoracic esophagectomy	RCT
14	Yang et al. [22]	2019	China	Multiple	360	– Robot‐assisted esophagectomy – Minimally invasive esophagectomy	RCT
15	Chao et al. [23]	2019	Taiwan, China	2	212	– Robot‐assisted esophagectomy – Video‐assisted thoracoscopic esophagectomy	RCT
16	Tagkalos et al [24]	2021	Germany, Netherlands, Switzerland	3	218	– Robot assisted minimally invasive oesophagectomy – Minimally invasive oesophagectomy	RCT
17	Li et al. [25]	2021	China	1	400	– Oesophagectomy with Two‐Field lymphadenectomy – Oesophagectomy with Three‐Field lymphadenectomy	RCT
18	Nuytens et al. [26]	2021	France	13	207	– Open oesophagectomy – Hybrid minimally invasive oesophagectomy	RCT
19	ROMIO Study Group [27]	2024	UK	Multiple	606	– Hybrid (laparoscopic/thoracotomic) – Open esophagectomy	RCT

Prospective cohort studies							
Study	Author	Publication year	Countries	Recruiting centres	Randomised patients	Interventions	Type of Study
PRE COS							
1	Hamouda et al. [28]	2010	UK	1	75	– Open Ivor Lewis oesophagectomy – Laparoscopic Ivor Lewis oesophagectomy	Prospective Cohort
2	Thomson et al. [29]	2010	Australia	1	221	– Open oesophagectomy – Minimally invasive oesophagectomy	Prospective Cohort
3	Willer et al. [30]	2010	USA	1	133	– Open trans‐thoracic oesophagectomy – Open trans‐hiatal oesophagectomy – Minimally invasive oesophagectomy	Prospective Cohort
4	Schoppmann et al. [31]	2010	Australia	1	62	– Open oesophagectomy – Minimally invasive oesophagectomy	Prospective Cohort
5	Safranek et al. [32]	2010	UK	1	118	– Open oesophagectomy – Minimally invasive oesophagectomy – Thoracoscopic hybrid oesophagectomy	Prospective Cohort
6	Pham et al. [33]	2010	USA	1	90	– Open Ivor–Lewis esophagectomy – Combined thoracoscopic‐laparoscopic esophagectomy	Prospective Cohort
7	Nafteux et al. [34]	2011	Belgium	1	175	– Minimally invasive oesophagectomy Open oesophagectomy	Prospective Cohort
8	Berger et al. [35]	2011	USA	1	118	– Minimally Invasive oesophagectomy – Open Ivor‐ Lewis oesophagectomy	Prospective Cohort
9	Blazeby et al. [36]	2011	UK	1	192	– Laparoscopically assisted oesophagectomy (LAO) – Three‐phase minimally invasive oesophagectomy (MIO) – Open two‐phase oesophagectomy	Prospective Cohort
10	Lee et al. [37]	2011	Taiwan	1	138	– Open oesophagectomy – Hybrid minimally invasive oesophagectomy – Total minimally invasive oesophagectomy	Prospective Cohort
11	Ovrebo et al. [38]	2012	Norway	1	147	– Transthoracic esophagectomy – Trans hiatal esophagectomy	Prospective Cohort
12	Parameswaran et al. [39]	2013	UK	2	97	– Open oesophagectomy – Laparoscopically assisted oesophagectomy – Totally minimally invasive oesophagectomy	Prospective Cohort
13	Yasuda et al. [40]	2013	Japan	1	20	– Two‐field lymphadenectomy (2FL) – Three‐field lymphadenectomy (3FL)	Prospective Cohort
14	Noble et al. [41]	2013	UK	1	106	– Totally minimally invasive – Open oesophagectomy – Open Ivor‐Lewis oesophagectomy	Prospective Cohort
15	Davies et al. [42]	2014	UK	1	644	– Transthoracic oesophagectomy – Hiatal oesophagectomy	Prospective Cohort
16	Ninomiya et al. [43]	2014	Japan	1	132	– Video‐assisted thoracoscopic radical esophagectomy (VATS) in the left lateral position	Prospective Cohort
17	Van der Sluis et al. [44]	2015	Netherlands	1	108	– Robot‐assisted thoraco‐laparoscopic esophagectomy – Open three‐stage transthoracic esophagectomy	Prospective Cohort
18	Zhao et al. [45]	2015	China	1	63	– Minimally invasive oesophagectomy – Open oesophagectomy	Prospective Cohort
19	Rinieri et al. [46]	2016	France	1	140	– Open oesophagectomy – Hybrid minimally invasive oesophagectomy	Prospective Cohort
20	Yu et al. [47]	2016	China	1	83	– Laparoscopic transhiatal esophagectomy (LTE) – Combined thoracoscopic and laparoscopic esophagectomy (CTLE)	Prospective Cohort
21	Deldycke et al. [48]	2016	Belgium	1	322	– Ivor Lewis esophagectomy (ILE) with intrathoracic anastomosis.	Prospective Cohort
22	Chan et al. [49]	2016	UK	1	50	– Endoscopic 2‐stage oesophagectomy (MIO) – Open oesophagectomy	Prospective Cohort
23	Park et al. [50]	2016	China	1	105	– Robot‐assisted esophagectomy – Thoracoscopic esophagectomy	Prospective Cohort
24	Nozaki et al. [51]	2017	Japan	37	379	– Open oesophagectomy – Thoracoscopic oesophagectomy	Prospective Cohort
25	Yao et al. [52]	2017	China	1	139	– Thoracoscopic‐laparoscopic esophagectomy (TLE) – Thoracoscopic esophagectomy (TE)	Prospective Cohort
26	Phillips et al. [3]	2017	UK	1	305	– A minimal abdominal lymphadenectomy – A minimal abdominal and thoracic lymphadenectomy – Exclusion of proximal thoracic nodes	Prospective Cohort
27	Charalabopoulos et al. [53]	2017	UK	1	13	– Minimally invasive oesophagectomy 2D thoracoscopic approach – Minimally invasive oesophagectomy 3D thoracoscopic approach	Prospective Cohort
POST COS							
28	Klevebro et al. [54]	2018	Sweden	1	366	– Open oesophagectomy – Minimally invasive oesophagectomy	Prospective Cohort
29	Motoyama et al. [55]	2019	Japan	1	59	– Robotic oesophagectomy – Minimally invasive oesophagectomy	Prospective Cohort
30	Sarkaria et al. [56]	2019	USA	1	170	– Robot‐assisted esophagectomy – Open oesophagectomy	Prospective Cohort
31	Lorenzi et al. [57]	2019	UK	1	40	– Hybrid (Laparoscopic/Thoracotomic) – Totally minimally invasive Ivor‐Lewis oesophagogastrectomy	Prospective Cohort
32	Deng et al. [58]	2019	China	1	151	– Robotic oesophagectomy – Minimally invasive oesophagectomy	Prospective Cohort
33	Yun et al. [59]	2020	Korea	1	371	– Robotic oesophagectomy – Open oesophagectomy	Prospective Cohort
34	Meredith et al. [60]	2020	USA	1	856	– Minimally invasive oesophagectomy – Open oesophagectomy	Prospective Cohort
35	Liu et al. [61]	2020	China	1	98	– Mediastinoscopy‐assisted trans hiatal esophagectomy – Thoraco‐laparoscopic esophagectomy	Prospective Cohort
36	Corsini et al. [62]	2021	USA	1	604	– Standard Ivor Lewis oesophagectomy – Modified en‐block Ivor Lewis oesophagectomy	Prospective Cohort
37	Fanfang Liu [63]	2023	China	2	5822	– Minimally Invasive Oesophagectomy – Open oesophagectomy	Prospective Cohort
38	Fanfang Liu [64]	2024	China	2	5556	– Right thoracic esophagectomy – Left thoracic esophagectomy	Prospective Cohort
39	Vercoulen [65]	2024	Netherlands	1	75	– Minimally Invasive transcervical Esophagectomy (MICE) – Open Esophagectomy	Prospective Cohort

There were 42 (72.4%) studies comparing minimally invasive surgery with open approaches over 15 years. These included robotic, laparoscopic, and hybrid (e.g., a combination of laparoscopic abdominal and open thoracic) procedures. Surgical approaches (e.g., transthoracic *vs.* transhiatal) were the next most commonly studied (18.9%).

### 3.3 Core Outcome Reporting

There were 9 RCTs and 27 prospective cohort studies pre‐publication of the COS. The number of RCTs and prospective cohort studies post‐publication was 10 and 12, respectively. No study reported all core outcomes of the COS. The average number of outcomes reported was 4 (interquartile range 3–5).

In addition to the COS, study outcomes included postoperative morbidity, fatigue, lung collapse, bile leak, chylothorax, multiorgan dysfunction syndrome and cerebrovascular events. Five studies reported single or no outcomes from the COS. These prioritised oncological outcomes such as 5‐year survival, disease‐free survival rates, R0 resection rate, number of resected lymph nodes (LNs) and patterns of recurrence.

Table 3 demonstrates the reporting ratio of outcomes of included studies pre and post‐publication of the COS. There were no outcomes of statistical significance in either group (Table 4).

**Table 3:** Subgroup analysis of all included studies.

RCT's				Prospective studies		
	Pre‐publication of COS in 2018 *n* = 9 (47.4%)	Post‐publication of COS in 2018 *n* = 10 (52.6%)	*p* value	Pre‐publication of COS in 2018 *n* = 27 (69.2%)	Post‐publication of COS in 2018 *n* = 12 (30.7%)	*p* value
**Overall survival**	2 (10.5% of RCT's)	6 (31.6% of RCTs)	0.18	7 (17.9% of Prospective studies)	0 (0% of Prospective studies)	0.31
**In‐hospital mortality**	9 (47.4%)	9 (47.4%)	0.42	23 (58.9%)	7 (17.9%)	1.00
**Overall quality of life**	3 (15.8%)	5 (26.3%)	0.18	3 (7.7%)	1 (2.6%)	1.00
**Need for additional intervention**	1 (5.2%)	2 (10.5%)	0.55	5 (12.8%)	2 (5.1%)	0.64
**Respiratory complications**	8 (42.1%)	9 (47.4%)	1.00	20 (51.3%)	6 (15.4%)	1.00
**Conduit necrosis and anastomotic leak**	8 (42.1%)	8 (42.1%)	0.57	22 (56.4%)	6 (15.4%)	1.00
**Severe nutritional problems**	3 (15.8%)	4 (21.1%)	0.37	1(2.6%)	1(2.6%)	0.36
**Ability to eat and drink**	5 (26.3%)	5 (26.3%)	1.00	10 (25.6%)	2 (5.1%)	0.69
**Acid indigestion or heartburn**	3 (15.8%)	4 (21.1%)	0.38	3 (7.7%)	0 (0%)	1.00
**Inoperability**	2 (10.5%)	5 (26.3%)	0.38	6 (15.4%)	1(2.6%)	1.00

*Note:* Subgroup analysis of studies pre and post publication of COS demonstrating no statistical significance.

**Table 4:** Defining and measuring outcomes.

	Studies Reporting outcome (*n*)	Studies providing definitions (*n*)	Measurement Tool	Timeframe
**Overall survival**	17 (29.8%)	6 (10.3%)	‐‐‐	2/3/5 years post‐operatively
**In‐hospital mortality**	49 (85.9%)	8 (13.8%)	‐‐‐	30/60/90 days post‐operatively
**Inoperability**	13 (22.8%)	3 (5.17%)	‐‐‐	‐‐‐
**Overall quality of life**	15 (26.3%)	‐‐‐	(Patient reported Outcome Measures questionnaire) SF‐36, CAT EORTC QLQ‐C30, CAT EORTC QLQ‐OES18, VAS, FACT‐E, HAD′s score, Fatigue score (MFI‐20), Modified Katz and the Lawton and Brody Scale	—
**Severe nutritional problems**	11 (19.3%)	8 (13.8%)	OES 18	
**Ability to eat and drink**	25 (43.9%)	8 (13.8%)	OES 18	
**Acid indigestion or heartburn**	12 (21.05%)	8 (13.8%)	OES 18	
**Respiratory complications**	45 (78.9%)	14 (24.1)	Esophagectomy Complications Consensus Group (‘ECCG′) classification (*n* = 2)	
**Conduit necrosis and anastomotic leak**	46 (80.7%)	‐‐‐	Esophagectomy Complications Consensus Group (‘ECCG′) classification (*n* = 2)	‐‐‐
**Need for additional intervention**	9 (15.8%)	‐‐‐	‐‐‐	30 days post‐operatively

*Note:* Overview of the definitions, measurement tools and their timeframe included in various studies.

### 3.4 Core Outcome Reporting Trends

**Figure 2:**
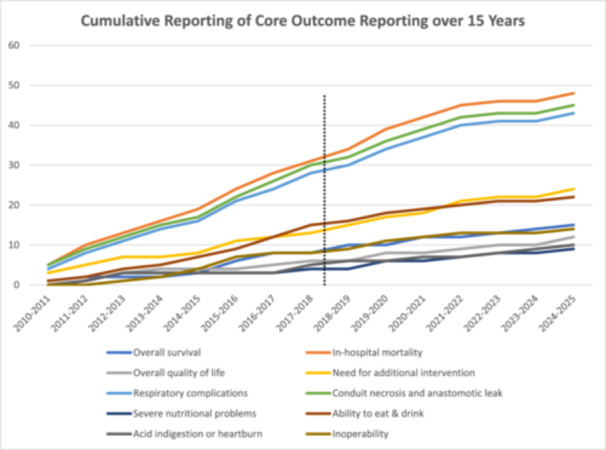
Cumulative trends in outcome reporting over the past 15 years demarcated by publication of COS in 2018.

No discernible trends were identified over the past 15 years. (Figure 2). The most consistently reported outcomes were surgery‐related death (86%), conduit necrosis and leak (81%) and respiratory complications (79%).

### 3.5 Defining and Measuring Outcomes

Thirty studies allocated a time period post‐operatively to report ‘in‐hospital mortality′. Three studies defined ‘inoperability′ as irresectability due to advanced disease found intra‐operatively. Twelve studies provided descriptions of the questionnaires given to patients to ascertain ‘overall quality of life′. These included a combination of several quality‐of‐life surveys, e.g. SF‐36, CAT EORTC QLQ‐C30, CAT EORTC QLQ‐OES18, VAS, FACT‐E and HAD′s score. Definitions for ‘respiratory complications varied from broad terminology such as ‘pneumonia,′ ‘atelectasis,′ and ‘pulmonary embolism′ to specific ones such as Computed Tomography (CT) and sputum culture‐proven pneumonia. The second most reported outcome was ‘conduit necrosis and leak′ (79%/*n* = 46), with two studies utilising the ECCG classification to define it. ‘Severe nutritional problems′ and ‘acid reflux and indigestion′ were the two least reported patient‐centred outcomes. Both were assessed via the OES 18 questionnaire, which included questions, such as, “Have you had trouble swallowing your own saliva?” and “Have you choked while swallowing?”. The ‘ability to eat and drink′ was recorded in 41.4% (*n* = 24) of studies. Nine studies used the EORTC QLQ‐OES18 questionnaire. The need for additional intervention, such as a return to theatre, was the lowest recorded surgical outcome.

### 3.6 Serious Adverse Events

Although not part of the COS, the majority (*n* = 54) of studies reported serious adverse outcomes. These were inconsistently defined. Twenty‐six studies used broad terms such as ‘complications′, ‘post‐operative complications′, or ‘intraoperative complications′. Four used terminology such as ‘morbidity′ or ‘general morbidity′. Some commented on individual outcomes (*n* = 12), while eight studies pooled complications based on severity or peri‐operative timing. Several studies (*n* = 27) reported adverse events as a combination of both.

Twenty‐three studies (39.7%) utilised grading systems such as Clavien‐Dindo, Common Terminology Criteria for Adverse Events, Comprehensive Complication Index, or criteria set by Low et al. [66].

## 4 Discussion

This review mapped the outcomes, described in a COS, for oesophageal cancer resection studies over the past 15 years and demonstrates the need for further standardisation of reporting in the literature. Overall, our study shows that there has been no significant improvement in the reporting of core outcomes since the COS was published in 2018.

Results show considerable variation in the priorities of oesophageal cancer resection studies globally.

Some studies have clearly defined objectives from the outset, focusing solely on oncological outcomes such as the extent of lymphadenectomy and resection margins. However, most studies reported outcomes related to short‐term complications. Similar findings were observed in the COMPAC (Core Outcome Measures for Perioperative and Anaesthetic Care) study, which aimed to establish core outcome sets in perioperative care and anaesthesia [67]. Patient‐reported outcomes were consistently poorly prioritised compared to clinical.

Selective reporting of certain outcomes leads to bias and an erosion of public trust in clinical studies. A significant proportion of planned study outcomes are not being reported as they may be subjectively deemed unimportant by researchers [68]. Preventing reporting bias and ensuring the representation of all stakeholders are compelling reasons for implementing a COS in the reporting of all research examining oesophagectomy practice.

The COS referenced in our study does not encompass serious adverse events and oncological outcomes. Calls for standardisation in the reporting of serious adverse events have been made by Low et al., [66] who formulated guidelines through a collaborative effort across 21 international centres. There is a rationale for developing distinct COS′ for oesophagectomies to categorise surgical, oncological, and patient‐centred outcomes. There is significant ambiguity in outcome definition across various studies, with endpoints and time periods being either undefined or unclear. The COMET (Core Outcome Measures in Effectiveness Trials) initiative launched in 2010 and the Standardising Endpoints for Perioperative Medicine (StEP) initiative in various medical disciplines aims to tackle the problem of loosely defined endpoints in surgical and oncological literature. It would complement the creation and implementation of a COS [9]. The reporting of a minimum number of core outcomes is critical to ensuring transparency in evaluating comparable trial data globally. It is a key factor in promoting equitable evidence synthesis and reducing research waste [69].

Finally, international consensus and the regular updating of a COS to guide future trials will be central to its utilisation. A UK‐based guideline may not be generalisable to the surgical or oncological needs in other demographic settings [69]. However, analysis of the GASTROS (COS for gastric cancer surgery) trial for gastric cancer demonstrated little disparity in the prioritisation of outcomes among international stakeholders, suggesting that core outcomes could similarly present a baseline for reporting expectations in oesophagectomy trials, except for a few that might be specific to region or country [70].

Ongoing and future RCTs may also provide scope for more up‐to‐date information to be disclosed, but we anticipate that improvement in the quality of reporting can be made only if there is concurrent uptake in the use of a COS. With a rise in the incidence of oesophageal carcinomas globally, the need to develop an international consensus on the outcomes of surgery to reliably share and analyse information in future studies becomes ever more crucial [71].

### 4.1 Strengths and Limitations

This review is the first of its kind comparing surgical studies in the field of oesophageal cancer treatment in the context of core outcome reporting. Its strength lies in the use of a consensus‐driven core outcome set together with a comparison of studies past and present, providing a comprehensive overview of this field.

The COS was primarily created to support the reporting of outcomes of RCTs. However, the inclusion of non‐randomised studies provides the breadth necessary for accurate representation of ongoing studies in this discipline. The first comprehensive multicentre RCT for oesophageal carcinoma resections was performed only 6 years ago [1]. The bulk of our review generally consisted of prospective cohort studies. This posed a challenge to sourcing high‐quality evidence from which to interpret results. Due to the high risk of morbidity and mortality, multicentre RCTs on oesophageal cancer resections face several ethical barriers to successful completion, especially when newer techniques and surgical modalities are being trialled. This is in addition to the investment of considerable time and resources. For this reason, we would not expect to see an immediate uptake of core outcome reporting in the period following its publication. Therefore our analysis of studies pre and post publication is merely indicative.

However, prospective studies can provide a snapshot of everyday clinical practice [63]. By improving the quality of non‐RCT comparative studies through standardisation, non‐randomised studies can still be beneficial in supplementing the results from RCTs to guide clinical practice [69].

The inclusion of English language studies alone may represent a limitation due to the high incidence and mortality associated with oesophageal cancer in Central and East Asia, with 90% incidence of the squamous cell type in these regions [72].

Finally, our review did not include multimodal treatment as the COS pertains primarily to surgical interventions undertaken. Surgical trials contain considerable heterogeneity in themselves and, therefore, were the focus of this study. Future work will endeavour to address both surgical and non‐surgical interventions.

## 5 Conclusion

Core outcomes following oesophagectomy that are of critical importance to healthcare professionals and patients are poorly reported in the literature. This picture has not improved over time. Further investment is required to increase the uptake of COS in oesophageal cancer research.

## Author Contributions

Bilal Alkhaffaf conceptualised this review. Kerry Avery, Natalie Blencowe, and Rhiannon Macefield provided the search strategy and studies included in previous reviews on the topic. Nadia Matias conducted search, abstract and full paper screening, data acquisition and analysis and wrote the first draft of the manuscript. Anie Naqvi, Jack Thomson and Roukia Techache assisted as second reviewers in abstract screening, full paper review and data acquisition. All authors provided critical comments and approved the final version of the manuscript.

## Funding

The authors received no specific funding for this work.

## Conflicts of Interest

The authors declare no conflicts of interest.

## Synopsis

This systematic review evaluates the extent to which core outcomes are reported in surgical trials and prospective studies of oesophageal cancer surgery. Among 58 eligible studies, no trial reported all 10 agreed‐upon core outcomes, with minimal improvement over time. These findings highlight the need for standardised outcome reporting to improve the quality and comparability of surgical research in oesophageal cancer.

## Supporting information

PRISMA 2020 checklist complete.

## Data Availability

Data sharing is not applicable to this article as no new data were generated or analysed in this study.

## Appendix 1

Search Strategy

*Database: Ovid MEDLINE*

1. Oesophagectomy {Including Related Terms}
2. exp Carcinoma, Squamous Cell/or exp Adenocarcinoma/or exp Esophagectomy/or exp Oesophageal Neoplasms/or exp Postoperative Complications/or exp Humans/
3. exp Carcinoma, Squamous Cell/or exp Adenocarcinoma/or exp Esophagectomy/or exp Oesophageal Neoplasms/or exp Postoperative Complications/or *Humans/
4. Oesophagect*.mp. [mp=title, abstract, original title, name of substance word, subject heading word, floating sub‐heading word, keyword heading word, organism supplementary concept word, protocol supplementary concept word, rare disease supplementary concept word, unique identifier, synonyms]
5. 3 or 4
6. 3 and 4
7. Neoplasms/
8. Cancer*.mp. [mp=title, abstract, original title, name of substance word, subject heading word, floating sub‐heading word, keyword heading word, organism supplementary concept word, protocol supplementary concept word, rare disease supplementary concept word, unique identifier, synonyms]
9. 7 or 8
10. 7 and 8
11. *Outcome Assessment, Health Care/or *Treatment Outcome/or exp *Humans/or exp Adult/or *Research Design/or exp Clinical Trials as Topic/
12. Core Outcom*.mp. [mp=title, abstract, original title, name of substance word, subject heading word, floating sub‐heading word, keyword heading word, organism supplementary concept word, protocol supplementary concept word, rare disease supplementary concept word, unique identifier, synonyms]
13. 11 or 12
14. exp Humans/or exp Adult/or *Observational Study/
15. exp “Systematic Review”/
16. exp Systematic Reviews as Topic/or exp Humans/or exp Randomised Controlled Trials as Topic/
17. Systematic Review*.mp. [mp=title, abstract, original title, name of substance word, subject heading word, floating sub‐heading word, keyword heading word, organism supplementary concept word, protocol supplementary concept word, rare disease supplementary concept word, unique identifier, synonyms]
18. 14 or 15 or 16 or 17
19. 5 and 9 and 13 and 18
20. limit 19 to (“all adult (19 plus years)” and english and meta analysis)
